# Susceptibility to pattern glare following stroke

**DOI:** 10.1007/s00415-012-6418-5

**Published:** 2012-01-31

**Authors:** Ian G. Beasley, Leon N. Davies

**Affiliations:** 1 Ophthalmic Research Group, Life and Health Sciences, Aston University, Birmingham, B4 7ET UK; 2 Aston Research Centre for Healthy Ageing (ARCHA), Aston University, Birmingham, UK

**Keywords:** Stroke, Pattern glare, Cortical hyperexcitability

## Abstract

The aim of this work was to measure susceptibility to pattern glare within a stroke group, employing a direct method of assessment. Twenty stroke subjects, aged 38–85 years, were recruited, along with an age-matched control group (*n* = 20). Assessment of pattern glare susceptibility was undertaken using the pattern glare test. An abnormal degree of pattern glare is present when individuals score >1 on the mid-high spatial frequency difference variable, a relative score that allows for normalization of the subject, or >3 when viewing the mid spatial frequency grating. Stroke subjects demonstrate elevated levels of pattern glare compared to normative data values and a control population, as determined using the pattern glare test. This was most notable when considering the output measure for the mid-high difference variable. The mean score for the mid-high difference variable was 2.15 SD 1.27 for the stroke subjects versus 0.10 SD 1.12 for the control subjects. When considering the mid-high difference variable, 75% of the stroke group recorded an abnormal level of pattern glare compared to 5% in the control group. This study demonstrates an association between stroke subjects and elevated levels of pattern glare. Cortical hyperexcitability has been shown to present following stroke, and this has been proposed as a plausible explanation for the perceptual distortions experienced by individuals susceptible to pattern glare. Further work to assess the benefits of spectral filters in reducing perceptual distortions in stroke patients is currently underway.

## Introduction

Ocular discomfort often manifests when viewing certain image types, such as repetitive striped patterns [1]. The intensity of these effects will vary according to individual susceptibility and the precise nature of the pattern, most notably, its spatial frequency and contrast level [2]. An adverse response when viewing stimuli of this type, i.e., striped patterns, has been termed ‘patterned glare’ [3] and latterly ‘pattern glare’ [4]. For susceptible individuals, this can result in visual stress, giving rise to symptoms of eyestrain, headaches and glare, along with illusions of colors, shapes, and motion [3]. The symptoms that arise from pattern glare susceptibility, resulting in visual stress, are often referred to by their historically derived terms, Meares–Irlen syndrome and scotopic sensitivity syndrome [5] and more recently referred to by the acronym MISViS, which denotes the title Meares–Irlen syndrome/visual stress [6]. The characteristics of the visual stimulus, which cause (or at least contribute in generating visual stress) is sensory in origin [7] and therefore distinguishable from factors of motor origin, such as oculomotor balance, binocular vergence, and accommodation [8]. The visual perceptual distortions that are generated by susceptibility to pattern glare is maximal when the spatial frequency of the stimulus is around three cycles per degree, with a pattern of even width and spacing, high contrast and viewed binocularly [1, 2]. Many of the attributes necessary to generate pattern glare in the susceptible individual are present within standard text documents formed by the individual rows of words separated by the successive spacing between rows [3, 9, 10]. The spatial frequency of this alternating high contrast pattern, formed by text, has been shown to fall within the range known to generate pattern glare symptoms [11]. This is coupled with striped patterns formed by letter strokes in individual words, as well as the vertical strokes of letters that also have a spatial frequency falling within a range sufficient to generate pattern glare symptoms in susceptible individuals. Although the origins of pattern glare and visual stress are equivocal, it is thought that these effects arise due to cortical hyper-excitability [2, 7, 12–14].

Pattern glare has been shown to be associated with a range of neurological conditions including photosensitive epilepsy [15], migraine [13, 16, 17], dyslexia [18–21], autism [22], multiple sclerosis [23], as well as MISViS [24, 25]. Hitherto, there is little evidence detailing the existence of pattern glare in other neurological conditions, such as stroke.

Some evidence has emerged from symptom-based investigation, where patients with traumatic brain injuries, including stroke, have demonstrated visual perceptual symptoms, which include light-sensitivity, strain, fatigue and reading difficulties which were subsequently reduced with the intervention of optimally selected tinted spectacle lenses [26]. Other reports have recognized a susceptibility to photophobia following traumatic brain injury and these individuals have shown improvements in reading ability with the use of photochromic filters [27]. Further evidence recognizes that many brain injury patients, including those with stroke, have difficulty with reading, such as skipping lines of text, and difficulty moving to the next row of print, albeit with the suggestion that these problems arise from motor rather than sensory origin [28]. It has been shown that even following minor head injuries, particularly those involving concussion, subjects demonstrate a lowered tolerance to brightness and sound [29]; this is supported by earlier evidence of photophobia and sound sensitivity in patients with closed head injury [30]. Measures of critical flicker frequency have also been found to be elevated in those with mild traumatic brain injury, with accompanying symptoms of light and motion sensitivity. It has been proposed that neurological disinhibition, as a result of the brain injury, may cause the subjective hypersensitivity to light and motion in these individuals [31]; this theory supports earlier work, which demonstrated hyperexcitability in the visual cortex, in patients with homonymous hemianopia, following stroke [32].

Stroke is the second leading cause of mortality, accounting for 9% of all deaths around the world, and is major cause of disability. The average age-adjusted stroke mortality for developed countries is around 50–100 per 100,000 people. With the burden of an ageing population, these figures are expected to increase greatly in the next two decades [33]. Considering the paucity of literature to date, it would seem logical to determine if there is an association between pattern glare and stroke. Consequently, the purpose of this study is to measure pattern glare susceptibility in stroke subjects as determined by the pattern glare test.

## Methods

Twenty stroke participants were recruited along with an equivalent number of age and gender-matched control subjects. Suitable candidates for this project were recruited by displaying notices at the research venue (the optometric practice of the investigator) requesting volunteers. Several participants were also recruited following contact with the local Stroke Association group. All candidates had been discharged from the care of the hospital department involved in the treatment of their stroke. The stroke participants consisted of 11 females and nine males with an age range of 38–85 years (mean 66.4 SD 13.43 years). The control participants had an age range of 36–84 years (mean 57.9, SD 13.91 years) consisting of 11 females and nine males. These groups were calculated as being age-matched (unpaired *t* test: *t* = 1.813, *df* = 38, *p* = 0.078). The time since the stroke (or most recent stroke) had a range of 0.83–12 years (mean 4.66, SD 3.31 years). Prior to commencing the research, ethical approval was obtained from Aston University’s Audiology/Optometry Research Ethics Committee with the study designed to follow the tenets of the Declaration of Helsinki. Each subject was given detailed information regarding the nature of the study, both verbally and in written form; this allowed informed consent to take place prior to their participation. The participants were required to complete a short questionnaire to ensure that they met the inclusion criteria. This was also necessary to pre-determine if any of the subjects had conditions that could potentially predispose them to pattern glare, e.g., migraine. Crucially, they were asked to confirm if they had suffered from any epileptic seizures, as this was the principal exclusion criterion for the study. Subjects were also asked to confirm if they were dyslexic, autistic, or suffered from multiple sclerosis, or migraine. All subjects demonstrated that they were able to read N8 sized print at 40 cm with their habitual spectacle correction, prior to undertaking the pattern glare test. From the initial questionnaires, two migraine sufferers were identified within the stroke participants and three migraine sufferers within the control subjects. None of the subjects in either group reported a diagnosis of dyslexia, autism, multiple sclerosis or epilepsy. One stroke participant (subject 2), had reported using a colored overlay and latterly, precision tinted lenses, on a regular basis, since having her stroke a few years prior, although these were not used whilst undertaking the pattern glare test.

The pattern glare test is designed to induce visual perceptual distortions in susceptible subjects and is now considered to be an established, efficient way to identify individuals with visual stress [6]. It is a simple test, consisting of three high-contrast gratings, each subtending 13.63° at the eye when viewed at 40 cm. Each pattern has a duty cycle of 50% with their grating orientation set horizontally to mimic text. The first pattern has a low spatial frequency (low-SF) of 0.3 cycles per degree (cpd), designed to trigger relatively few distortions and unlikely to have an association with headaches and eyestrain [3]; this first pattern, therefore, serves as a useful control for suggestive individuals. The second pattern is designed to elicit maximum distortions, with a mid-spatial frequency (mid-SF) of 2.3 cpd and those who respond adversely to this pattern are likely to experience symptoms of visual stress in their everyday environment. The third high-contrast grating serves as another control with a higher spatial frequency (high-SF) of 9.4 cpd and it would be expected that this would generate fewer symptoms than the second pattern. The patterns are viewed in succession and the numbers of symptoms generated are summed to give a pattern glare score for each of the gratings. Pattern glare would be suggested if the patient responds with a high score on the mid-SF grating and/or a score with the mid-SF pattern, which is greater that the score with the high-SF grating, the so-called ‘mid-high difference’ variable. This relative, rather than absolute, mid-high difference measure allows for normalization of the subject by accounting for suggestive individuals. Normative values for the pattern glare test have been established [6], taking into account that only a high test score should be regarded as clinically significant [34]. The normal range for the mid-SF pattern has been shown as a pattern glare score of <4, and an upper limit of the normal range for the mid-high difference variable as being 1. In other words, those with a pattern glare test Score of >3 on the mid-SF grating, or a score of >1 on the mid-high difference variable demonstrate an abnormal degree of pattern glare. The literature also shows that individuals with relatively low visual discomfort are more likely to report distortions with the high-SF than the mid-SF grating; this is attributable to a greater influence of optical factors when viewing this finer grating rather than symptoms of neurological origin. These individuals are also more likely to report fewer distortions overall [35]. It has been shown that pattern glare scores are similar in males and females. When considering age, younger participants were found to report more distortions on the mid-SF and the high-SF gratings than older subjects. Overall, with advancing age, there was found to be a greater decrease with the high-SF grating than the mid-SF grating which in turn, results in a small overall increase in the ‘mid-high difference’ variable, although this was not found to be of statistical significance. Migraine sufferers were found to score similarly to a control population when viewing each grating in turn, however they were found to score significantly higher when considering the mid-high difference variable [6].

All subjects were asked to perform the pattern glare test in line with the developer’s instructions [34]. The subjects were asked to view pattern 1 at 40 cm (low-SF), concentrating on the central square in the pattern for 5 s. They were then asked to report if any of the following effects were noticed: (1) colors, (2) bending of lines, (3) blurring of lines, (4) shimmering or flickering, (5) fading, (6) shadowy shapes (7) any other effects (to be specified). The subjects were also asked to specify if any of these effects were more noticeable mainly on the left side, right side or both sides of the pattern. The process was then repeated for pattern 2 (mid-SF) and finally for pattern 3 (high-SF). The numbers of symptoms reported were then added to give an individual pattern glare score for each of the three patterns. The scores for the high-SF grating were subtracted from the mid-SF grating to determine the mid-high difference variable.

### Statistical analysis

All data were analyzed using the commercially available software (PASW, *v*. 18, IBM, New York, USA). Data distributions were evaluated using the Kolmogorov–Smirnov test. A significance level of α = 0.05 was used throughout the analysis.

## Results

A summary of the data from the questionnaire and pattern glare test for the stroke and control participants can be seen in Tables 1 and 2, respectively.

**Table 1:** Data from questionnaire and results from pattern glare test for stroke subjects

Stroke subject	Age	Gender	Detail	PGS mid-SF	PGS mid-high difference	PGS high-SF
1	83	Female		5	5	0
2	38	Female	Precision tint	6	2	4
3	64	Female		5	3	2
4	72	Male		1	1	0
5	71	Male		5	3	2
6	52	Male		5	4	1
7	77	Female		3	2	1
8	65	Male		2	2	0
9	62	Male		3	2	1
10	69	Female		0	0	0
11	56	Female	Migraine	2	2	0
12	49	Female		3	3	0
13	76	Female		1	1	0
14	43	Male		4	2	2
15	81	Female		1	1	0
16	80	Female		5	4	1
17	74	Female		3	2	1
18	72	Male	Migraine	2	2	0
19	59	Male		0	0	0
20	85	Male		2	2	0

**Table 2:** Data from questionnaire and results from pattern glare test for control subjects

Control subject	Age	Gender	Detail	PGS mid-SF	PGS mid-high difference	PGS high-SF
1	45	Female	Migraine	1	1	0
2	38	Male		2	0	2
3	63	Male		0	−1	1
4	68	Female		0	0	0
5	70	Female		0	−2	2
6	55	Female		1	−1	2
7	84	Female		2	0	2
8	36	Female	Migraine	1	1	0
9	38	Male		2	1	1
10	62	Female	Migraine	1	1	0
11	55	Male		0	−1	1
12	60	Male		3	1	2
13	50	Male		3	−2	5
14	68	Female		1	−1	2
15	83	Male		1	1	0
16	61	Male		1	1	0
17	60	Male		4	1	3
18	54	Female		4	0	4
19	40	Female		4	2	2
20	78	Female		2	0	2

Data for the mid-SF pattern were found to be normally distributed for the stroke participants (*p* = 0.579) and also for the control subjects (*p* = 0.219). For the relative score of the mid-high cpd difference, the data were also normally distributed for the stroke subjects (*p* = 0.174) as well as the control participants (*p* = 0.202). Data for the stroke participants’ responses to the high-SF pattern were not found to exhibit a normal distribution (*p* = 0.045). However, for the control subjects these data were normally distributed (*p* = 0.271).

**Fig. 1:**
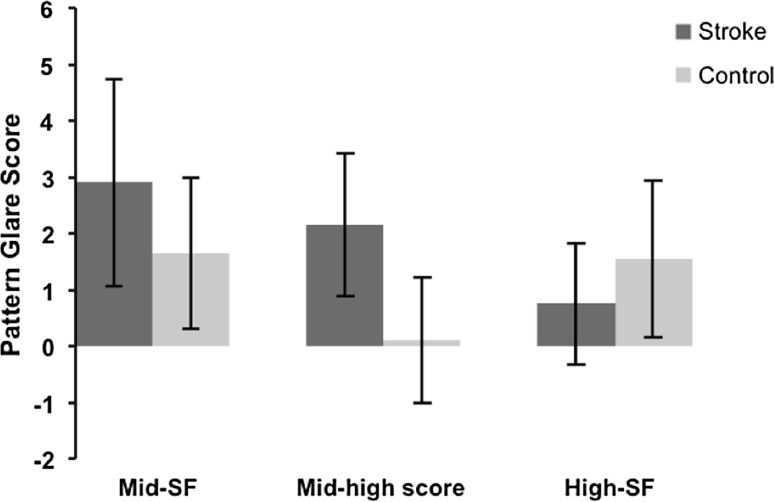
Mean pattern glare scores, from the pattern glare test, for stroke and control participants. Statistically significant elevation of pattern glare scores were found for the stroke subjects when considering the mid-SF grating, and most notably, the mid-high difference variable

Statistical analysis of the pattern glare scores (see Table 3; Fig. 1) showed significantly higher values for the stroke participants versus control subjects when viewing the mid-SF pattern (unpaired *t* test: *t* = 2.457, *df* = 38, *p* = 0.019); this was also the case when comparing the relative score of the mid-high difference (unpaired *t* test: *t* = 5.421, *df* = 38, *p* < 0.0001). When subjects were asked to view the high-SF spatial frequency grating, however, the control subjects yielded a higher pattern glare score than the stroke subjects (Mann–Whitney *U* test: *p* = 0.040).

**Table 3:** Results from the pattern glare test detailing mean pattern glare scores for stroke and control participants

	Score (mean ± SD)	Score (mean ± SD)
Mid-SF	2.9 ± 1.83	1.65 ± 1.35
Mid-high difference	2.15 ± 1.27	0.10 ± 1.12
High-SF	0.75 ± 1.07	1.55 ± 1.39
	Stroke (*n* = 20)	Control (*n* = 20)

One of the stroke participants (subject 2) had reported during the initial questionnaire that she had previously used spectral filters and continued to use precision tinted lenses on an on-going basis. Although these were not worn whilst undertaking the pattern glare test, the prolonged use of filters may have potentially altered performance on a test designed to induce visual stress. It is of interest to highlight, however, that this subject did score highly when viewing the mid-SF pattern (pattern glare score = 6), and moderately so on the relative score of the mid-high difference (pattern glare score = 2). Notwithstanding this, the data were considered again, with this subject omitted from all subsequent analyses. With the removal of this subject, the mean age of the stroke group (mean 67.89, SD 11.97 years) was higher than the control participants (mean 58.40, SD 14.46 years; unpaired *t* test: *t* = 2.227, *df* = 37, *p* = 0.032). This new data set were found to follow a normal distribution for scores on the mid-SF grating (*p* = 0.656) as well as for the relative score mid-high difference (*p* = 0.256) but not when considering data for the high-SF grating (*p* = 0.017).

**Fig. 2:**
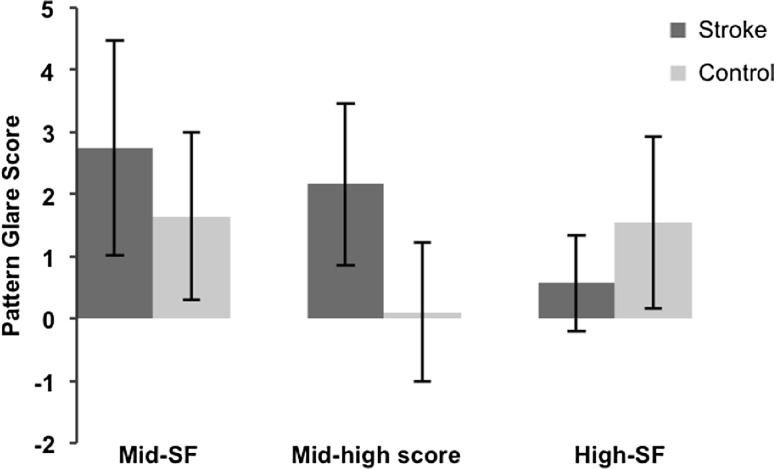
Mean pattern glare scores, from the pattern glare test, for stroke and control participants, with the exclusion of stroke subject 2

These data (see Table 4; Fig. 2), were comparable with earlier measures, demonstrating that the stroke participants score more highly on the mid-SF grating (unpaired *t* test: *t* = 2.197, *df* = 37, *p* = 0.034), and the mid-high difference variable (unpaired *t* test: *t* = 5.301, *df* = 37, *p* = 0.000), whereas they score lower on the high-SF grating than control subjects (Mann–Whitney *U* test: *p* = 0.016).

**Table 4:** Results from the pattern glare test detailing mean pattern glare scores for stroke and control participants, with the exclusion of subject 2 from the stroke group

Spatial frequency	Score (mean ± SD)	Score (mean ± SD)
Mid-SF	2.74 ± 1.73	1.65 ± 1.35
Mid-high difference	2.16 ± 1.30	0.10 ± 1.12
High-SF	0.58 ± 0.77	1.55 ± 1.39
	Stroke (*n* = 19)	Control (*n* = 20)

The data were also considered for the effect of gender in the stroke group. The males (mean 64.56, SD 12.40 years) in the stroke group were slightly younger than the females (mean 70.90, SD 11.34 years), (unpaired *t* test: *t* = 1.165, *df* = 17, *p* = 0.260). The data were found to follow a normal distribution for the males when viewing the mid-SF pattern (*p* = 0.842), and for the mid-high difference variable (*p* = 0.491), as well as for the high-SF grating (*p* = 0.265). The data were also found to be normally distributed for the females on the mid-SF grating (*p* = 0.874), the mid-high difference variable (*p* = 0.904), and the high-SF pattern (*p* = 0.149).

**Fig. 3:**
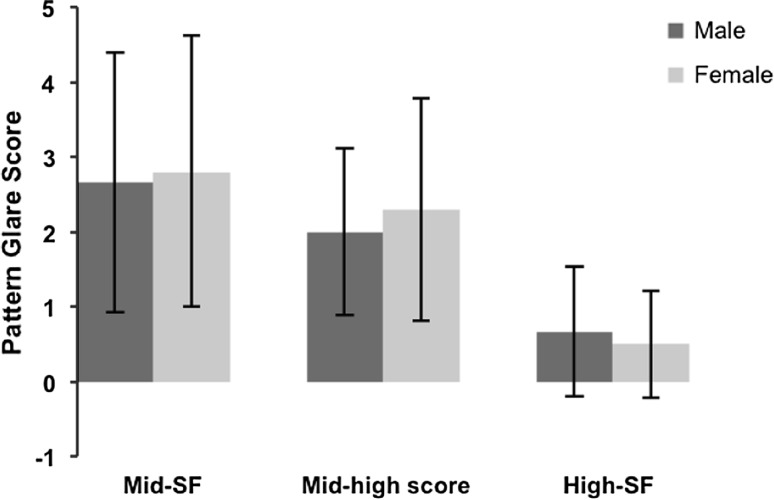
Mean pattern glare scores from the pattern glare test for male and female stroke participants. Results showed no statistically significant difference between the gender types for any of the measures

The results (see Table 5; Fig. 3), revealed a marginally higher pattern glare score for the females compared to the males when viewing the mid-SF grating (unpaired *t* test: *t* = −0.163, *df* = 17, *p* = 0.872). The females also demonstrated a higher mid-high difference variable score than the males, (unpaired *t* test: *t* = −0.491, *df* = 17, *p* = 0.630). Conversely, the males recorded a higher score when viewing the high-SF pattern (unpaired *t* test: *t* = −0.462, *df* = 17, *p* = 0.650).

**Table 5:** Results from the pattern glare test detailing mean pattern glare scores for gender comparison within the stroke group

Spatial frequency	Score (mean ± SD)	Score (mean ± SD)
Mid-SF	2.66 ± 1.73	2.80 ± 1.81
Mid-high difference	2.00 ± 1.11	2.30 ± 1.49
High-SF	0.66 ± 0.87	0.50 ± 0.71
	Male (*n* = 9)	Female (*n* = 10)

As pattern glare has a known association with migraine [13], the data were examined with the respective migraine sufferers in each group removed from the analysis. These groups were found to be age-matched for the stroke group (mean 68.35, SD 12.29 years) and the control group (mean 60.29, SD 14.18 years; unpaired *t* test: *t* = 1.770, *df* = 32, *p* = 0.086). The data for the stroke group with the migraine sufferers removed, were found to be normally distributed for the mid-SF pattern (*p* = 0.644), the mid-high difference variable (*p* = 0.518) and the high-SF grating (*p* = 0.056); this was also the case for the control group for the mid-SF pattern (*p* = 0.687), the mid-high difference variable (*p* = 0.671), and the high-SF grating (*p* = 0.165).

**Fig. 4:**
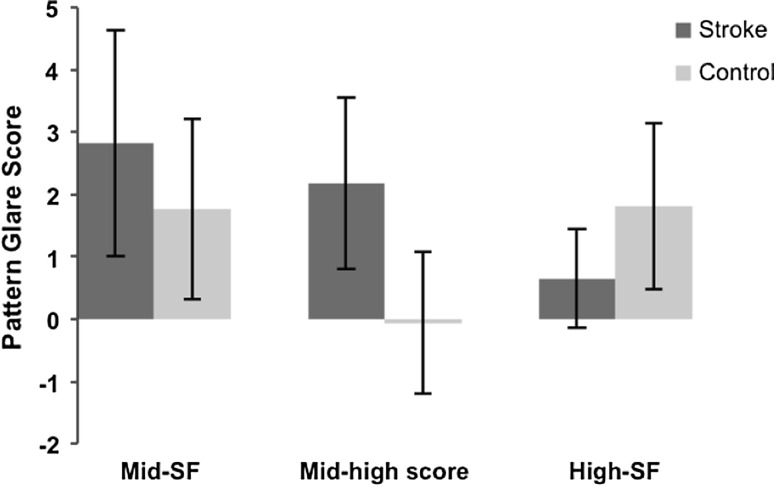
Mean pattern glare scores from the pattern glare test for stroke and control participants, with the exclusion of migraine subjects. Statistically significant elevation of pattern glare scores were found for the stroke subjects, when considering the mid-high difference variable

The results for this modified data set (see Table 6; Fig. 4), showed that the stroke subjects exhibited higher pattern glare scores than the control subjects when viewing the mid-SF grating (unpaired *t* test: *t* = 1.888, *df* = 32, *p* = 0.068), as well as for the relative score of the mid-high difference variable (unpaired *t* test: *t* = 5.141, *df* = 32, *p* = 0.0000). Consistent with earlier measures, the stroke subjects scored much lower on the pattern glare test, when viewing the high-SF pattern, compared to the control group (unpaired *t* test: *t* = −3.133, *df* = 32, *p* = 0.004).

**Table 6:** Results from the pattern glare test detailing mean pattern glare scores for stroke and control participants, with the exclusion of migraine subjects

Spatial frequency	Score (mean ± SD)	Score (mean ± SD)
Mid-SF	2.82 ± 1.81	1.76 ± 1.44
Mid-high difference	2.18 ± 1.38	−0.06 ± 1.14
High-SF	0.65 ± 0.79	1.82 ± 1.33
	Stroke (*n* = 17)	Control (*n* = 17)

## Discussion

The present study has demonstrated that stroke subjects respond with significantly higher pattern glare scores than a control population when undertaking the pattern glare test; this elevation in scores being most notable for the mid-high difference variable, a relative score that allows for normalization of the subject by taking into account suggestibility. Abnormal pattern glare is considered to be present when a subject delivers a pattern glare score of >3 on the mid-SF grating, or a score of >1 on the mid-high difference variable. In fact, 75% of the stroke participants recorded an abnormal degree of pattern glare when considering the mid-high difference measure, compared to just 5% within the control population. For the stroke participants, the mean score approached the cut-off for abnormality of >3 when viewing the mid-SF grating, but did not exceed it. It was, however, significantly higher than the scores of a control group, at a level of statistical significance. Further suggestion of elevated pattern glare in the stroke group was shown by a decreased measure on the high-SF grating for stroke subjects versus control subjects. The high-SF measure, which was found to be of statistical significance, is usually higher in subjects with a low degree of visual discomfort. These three principal measures were still found to persist even when subjects with potential correlates of pattern glare, namely migraine, were removed from the analyses. It should be noted that the control group were some 10 years younger than the stroke subjects. Established normative values have shown that with advancing age, there is a greater decrease with the high-SF grating than the mid-SF grating which, in turn, results in a small overall increase in the ‘mid-high difference’ variable, although not at levels of statistical significance [6].

Despite a paucity of literature, there is evidence of sensitivity to patterns following traumatic brain injury, including stroke, as determined by analyzing self-test responses to a questionnaire designed to recognize sensitivity to glare, light, contrast, bright colors, and patterns [26]. It has also been shown, however, that this indirect method of determining the existence of pattern related visual stress is not as useful as a direct presentation of a patterned stimulus to the individual concerned [36]. Whilst an indirect method of analyzing pattern glare symptoms serves as a useful indicator, it does not appear to isolate those individuals who are more likely to have their symptoms alleviated by an intervention such as a spectral filter.

Since pattern glare results from cortical hyperexcitability [7, 11], it is perhaps unsurprising that stroke sufferers can be shown to demonstrate abnormal levels of pattern glare; this is in line with evidence of cortical hyperexcitability following stroke [32]. Lesion-induced cortical hyperexcitability has also been demonstrated following short transient ischemic attacks [37], suggesting that symptoms of pattern glare could potentially exist in those experiencing milder episodes of so-called ‘mini-stroke’ in the absence of more obvious functional impairment, and represents an opportunity for further work in this area. It would be valuable for future studies to consider the association of pattern glare, with co-existing visual deficits, such as visual field defects and binocular vision anomalies, in stroke individuals.

The present study was limited by a modest sample size, and further work with a larger subject group should be considered, to examine pattern glare trends in a broader stroke population. Furthermore, it should be recognized that some of the sample were recruited from an optometric practice, and given that these subjects may have a higher predisposition to visual symptoms associated with their stroke, this could overestimate the levels of visual stress in stroke patients. An opportunity for further work would be to consider the effect of time interval, and number of stroke events, on the outcome measures. In the present study, the majority of the subjects had suffered a stroke several years prior to the data collection.

In conclusion, it appears that pattern glare is associated with stroke, indicated by an abnormal pattern glare score on the pattern glare test, most notably when considering the mid-high difference variable. This remains the case when those with other co-morbidities of pattern glare, such as migraine, or previously identified visual stress, are removed from the data set. Further work is required to investigate other measures of visual perceptual deficits within a stroke group, along with the management of these symptoms with spectral filters.
